# TRPM5 rs886277 Polymorphism Predicts Hepatic Fibrosis Progression in Non-Cirrhotic HCV-Infected Patients

**DOI:** 10.3390/jcm10030483

**Published:** 2021-01-28

**Authors:** Salvador Resino, Amanda Fernández-Rodríguez, Daniel Pineda-Tenor, Ana Zaida Gómez-Moreno, Juan José Sánchez-Ruano, Tomas Artaza-Varasa, María José Muñoz-Gómez, Ana Virseda-Berdices, María Martín-Vicente, Isidoro Martínez, María A. Jiménez-Sousa

**Affiliations:** 1 Unidad de Infección Viral e Inmunidad, Centro Nacional de Microbiología, Instituto de Salud Carlos III, 28222 Majadahonda, Spain; amandafr@isciii.es (A.F.-R.); mjose.munoz@isciii.es (M.J.M.-G.); anavirseda@isciii.es (A.V.-B.); maria.martinv@externos.isciii.es (M.M.-V.); imago@isciii.es (I.M.); jimenezsousa@isciii.es (M.A.J.-S.); 2 Servicio de Laboratorio Clínico, Hospital de Antequera, 29200 Málaga, Spain; dpinedatenor@gmail.com; 3 Servicio de Digestivo, Hospital Virgen de la Salud, 45004 Toledo, Spain; ana.zaidag@hotmail.com (A.Z.G.-M.); jjsanchezr@sescam.jccm.es (J.J.S.-R.); tdeartaza@gmail.com (T.A.-V.)

**Keywords:** chronic hepatitis C, hepatic fibrosis, cirrhosis, liver stiffness, TRPM5, SNPs

## Abstract

Background: *TRPM5* (transient receptor potential cation channel subfamily M member 5) rs886277 polymorphism has been related to liver cirrhosis from different etiologies. The present study investigates the association of *TRPM5* rs886277 polymorphism with liver fibrosis progression and cirrhosis development in chronic hepatitis C (CHC) patients. Methods: We conducted a retrospective study of 208 non-cirrhotic patients with CHC, who had at least two liver stiffness measurements (LSM) with a separation of 12 months (baseline LSM (LSM1) and the last LSM (LSM2)). Two outcome variables were considered: (1) LSM2/LSM1 ratio; (2) cirrhosis progression (F4; LSM ≥ 12.5 kPa). DNA genotyping was done at the CeGen using a MassARRAY platform. Results: The follow-up time was similar irrespective of the rs886277 genotype (46.4 months in TT genotype, 46.4 months in CT genotype, and 49.2 months in CC genotype; *p* = 0.649). The highest LSM increases were found in patients with CC genotype compared with TT and CT genotypes (*p* = 0.044 and *p* = 0.038, respectively). The cirrhosis progression was higher in patients with CC genotype than TT genotype (*p* = 0.033). Thus, the rs886277 C allele was associated with higher cirrhosis progression (adjusted odds ratio (aOR) = 2.64; *p* = 0.014). Moreover, rs886277 CC genotype was also related to higher values of LSM2/LSM1 ratio (adjusted arithmetic mean ratio a(AMR) = 1.31; *p* = 0.001) and cirrhosis progression (aOR = 4.33; *p* = 0.027). Conclusions: *TRPM5* rs886277 polymorphism was associated with liver fibrosis progression and cirrhosis development among hepatitis C virus (HCV)-infected patients. Specifically, the rs886277 C allele and CC genotype were risk factors for advancing liver fibrosis and cirrhosis compared to the rs886277 T allele and CT/TT genotype, respectively.

## 1. Introduction

Chronic hepatitis C (CHC) remains a significant public health problem worldwide. About 71 million people are chronically infected with the hepatitis C virus (HCV), and CHC is one of the leading causes of liver-related death and disability worldwide [1,2,3]. Patients develop hepatic fibrosis, cirrhosis, decompensated cirrhosis, hepatic failure, and hepatocarcinoma after decades of infection [4]. Even patients who clear HCV infection after treatment with direct-acting antivirals (DAAs) remain at risk of liver disease progression, mostly cirrhotic patients [5,6].

The staging of hepatic fibrosis helps in the clinical management of patients with CHC and may predict its evolution [7]. The hepatic biopsy is the gold standard to assess liver fibrosis, but this practice is in disuse for its contraindications and limitations, such as invasive procedure, sampling errors, reading variability, hospitalization, cost, and delayed results, among others [8]. Transient elastography is a non-invasive approach widely used to evaluate liver fibrosis and cirrhosis [9]. The transient elastography quantifies liver stiffness, which is proportional to the grade of hepatic fibrosis and correlates with fibrosis stage in CHC [10]. However, transient elastography also has limitations such as variability, inadequate accuracy, and risk for error [11].

Many factors are implicated in liver disease progression, such as transmission routes, age at HCV infection, alcohol intake, duration of CHC, coinfections, insulin resistance, and steatosis [4]. Furthermore, the patient’s genetic background, including single nucleotide polymorphisms (SNPs), appears to be quite relevant in CHC pathogenesis and cirrhosis progression [12,13].

The transient receptor potential cation channel subfamily M member 5 (TRPM5) gene encodes a calcium-activated non-selective cation channel that participates in the signaling mechanism for the taste sensation and insulin secretion in pancreatic β-cells [14,15]. Furthermore, TRPM5 is involved in the immune and inflammatory responses to different pathogens through the taste transduction pathway [16,17,18,19,20,21,22]. The *TRPM5* rs886277 polymorphism is a missense (Asn235Ser) variant related to liver fibrosis in HCV-infected patients, primarily as part of the cirrhosis risk score (CRS), which comprised seven SNPs predictive of fibrosis progression in HCV-infected patients [23,24,25,26,27,28] and liver transplantation [29]. However, most of these studies did not analyze cirrhosis progression, or rs886277 polymorphism was not directly associated with fibrosis or cirrhosis progression.

The present study’s objective was to investigate the association of *TRPM5* rs886277 polymorphism with liver fibrosis progression and cirrhosis development in CHC patients.

## 2. Methods

### 2.1. Design and Study Population

We conducted a retrospective study of 208 CHC patients from Hospital Virgen de la Salud (Toledo, Spain) enrolled between 2008 and 2016, as previously described [30]. The study was performed according to the 1975 Declaration of Helsinki, and the Research Ethics Committee of the Hospital Virgen de la Salud approved it (CEIC/2013/32). All the participants signed written consent.

The inclusion criteria were: (1) detectable plasma HCV RNA at baseline and during the whole follow-up; (2) available DNA sample for DNA genotyping; and (3) available data from liver stiffness measurements (LSM) at baseline and at least 12 months later. The exclusion criteria were: (1) baseline hepatic cirrhosis (F4; LSM1 ≥12.5 kPa); (2) autoimmune liver disease; and (3) coinfection with hepatitis B virus or human immunodeficiency virus. All patients were of European descent.

We collected epidemiological, demographic, clinical, virological, and laboratory data from medical records. The patients’ clinical management was done following clinical guidelines at that time [31,32]. All patients were CHC at baseline, including those who had been non-responder patients to interferon (IFN) therapy before the study. The follow-up was interrupted when a patient started the HCV treatment and achieved sustained virologic response (SVR). Patients who did not achieve SVR were not excluded from the study.

### 2.2. DNA Genotyping

We extracted genomic DNA from 200 µL of peripheral blood using the QIAsymphony DNA Mini Kit (Qiagen, Hilden, Germany). Next, DNA genotyping was done at the CeGen (Spanish National Genotyping Center; [33]) using the MassARRAY platform from Agena Bioscience’s (San Diego, CA, USA) [34].

### 2.3. Hepatic Fibrosis

Transient elastography was used to assay the hepatic fibrosis using a FibroScan (Echosens, Paris, France) by a trained physician, as we previously described [35]. LSM ranged from 2.5–75 kPa. Typically, around ten individual successful measurements were obtained and averaged when the interquartile range to median ratio was <0.30. The LSM cut-offs proposed by Castera et al. [36] were used to classify patients: (1) <7.1 kPa (F0–F1—absence or mild fibrosis); (2) 7.1–9.4 kPa (F2—significant fibrosis); (3) 9.5–12.4 kPa (F3—advanced fibrosis); and (4) ≥12.5 kPa (F4—cirrhosis).

### 2.4. Liver Fibrosis Outcomes

Each patient’s LSM value changed from the baseline LSM (LSM1) to the last LSM (LSM2) in the absence of successful antiviral treatment that cleared HCV infection. Thus, we consider three outcome variables: (1) Values of LSM in the two time-points (LSM1 and LSM2), (2) LSM2/LSM1 ratio, and (3) cirrhosis progression (F4; LSM ≥ 12.5 kPa).

### 2.5. HCV Assays

HCV infection was diagnosed by enzyme-linked immunosorbent assays and polymerase chain reaction (PCR) tests. HCV genotype was determined by the INNO–LiPA HCV II assay (Innogenetics, Ghent, Belgium). Plasma HCV RNA viral load was measured by real-time PCR COBAS AmpliPrep/COBAS TaqMan HCV test (Roche Molecular Systems, Pleasanton, CA, USA) and the limit of detection was 15 IU/mL.

### 2.6. Statistical Analysis

To compare independent groups, we used the Mann–Whitney U test and the Kruskal–Wallis test for continuous variables. In addition, the Chi-square test or Fisher’s exact test were used for categorical variables. In paired measurements, we used the Wilcoxon test for continuous variables.

The genetic association study between *TRPM5* rs886277 polymorphism and the outcome variables was performed by generalized linear models (GLM) for recessive, dominant, and additive inheritance. A GLM with gamma distribution was used to evaluate the LSM2/LSM1 ratio, which provides the arithmetic mean ratio (AMR). A GLM with binomial distribution was used to analyze cirrhosis progression, which provides the odds ratio (OR). GLM tests were adjusted by the most relevant covariables selected by a stepwise algorithm (*p*-value < 0.20 at each step) from the following list of variables: age, gender, time since HCV diagnosis, diabetes, injection drug use, high alcohol intake, time of follow-up, baseline LSM, HCV treatment (before and after starting the study among non-responder patients), HCV genotype, and other significant SNPs previously analyzed in this cohort (MERTK rs4374383 [37], PNPLA3 rs738409 [38], IL7RA rs6897932 [35], MTHFR rs1801133 [39], and DARC rs12075 [30]).

The statistical analysis was done with Stata 15.0 (StataCorp, TX, USA) and SPSS 24.0 (SPSS INC, Chicago, IL, USA). A *p*-value < 0.05 was statistically significant, and all *p*-values were two-tailed.

## 3. Results

### 3.1. Characteristics of the Patients

The characteristics of HCV-infected patients stratified by *TRPM5* rs886277 genotypes (85 TT, 95 CT, and 28 CC) are described in Table 1. We did not find significant differences in baseline characteristics among groups, except for HCV genotype 1 (*p* = 0.032) and 4 (*p* = 0.035).

### 3.2. Characteristics of TRPM5 rs886277 Polymorphism

Rs886277 SNP was in Hardy–Weinberg equilibrium (*p* = 0.858), had less than 5% of missing values, and had a minimum allele frequency of more than 35% (Table 2). When we compared the genetic frequencies of our cohort of HCV-infected patients with an Iberian population in Spain (IBS; healthy subjects) reported by the 1000 Genomes Project [40], no significant differences were found for alleles (*p* = 0.367) or genotypes (*p* = 0.816).

### 3.3. TRPM5 rs886277 SNP and Liver Fibrosis Progression

**Figure 1:**
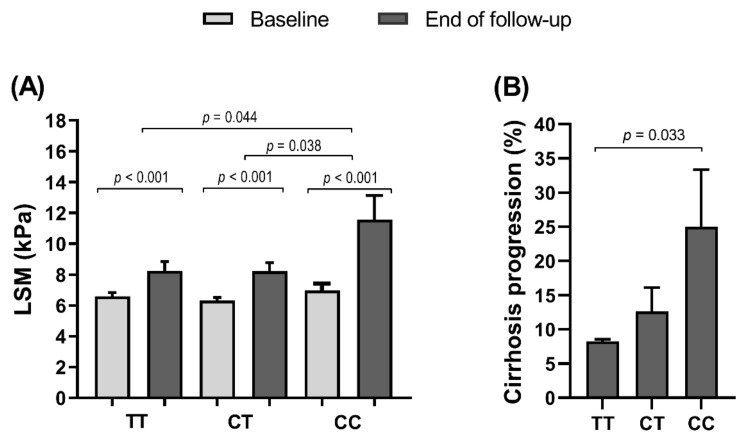
Summary of LSM values (**A**) and cirrhosis progression (**B**) stratified by *TRPM5* rs886277 genotypes in HCV-infected patients. Abbreviations: HCV, hepatitis C virus; LSM, liver stiffness measurement; IFN, interferon; *TRPM5*, transient receptor potential cation channel subfamily M member 5.

The follow-up time was similar among *TRPM5* rs886277 genotypes (46.4 months in TT genotype, 46.4 months in CT genotype, and 49.2 months in CC genotype; *p* = 0.649). Throughout this time, we found significant increases in LSM values at the end of follow-up within each rs886277 genotype, compared to baseline (*p* < 0.001; Figure 1A). However, the highest LSM increases were found in patients with CC genotype compared with TT and CT genotypes (*p* = 0.044 and *p* = 0.038, respectively). Similarly, the rate of cirrhosis progression was higher in patients with CC genotype than TT genotype (*p* = 0.033; Figure 1B).

We also evaluated the association between *TRPM5* rs886277 polymorphism and liver fibrosis progression by GLM tests (Table 3). Regarding the additive model, the presence of rs886277 C allele was associated with higher values of LSM2/LSM1 ratio (AMR = 1.15; *p* = 0.002) and cirrhosis progression (OR = 1.91; *p* = 0.032), but only cirrhosis progression remained significant after adjusting for the most relevant covariables (adjusted OR = 2.64; *p* = 0.014). That is, for each C allele, the risk of progressing to cirrhosis increases 2.64 times. With regard to the recessive model, rs886277 CC genotype was related to higher values of LSM2/LSM1 ratio (adjusted AMR = 1.31; *p* = 0.001) and cirrhosis progression (adjusted OR = 4.33; *p* = 0.027, Table 3). The presence of the CC genotype is associated with a 1.33-fold increase in the baseline LSM value and increases the risk of progressing to cirrhosis 4.33 times.

## 4. Discussion

This study focused on the impact of *TRPM5* rs886277 polymorphism on liver fibrosis progression and cirrhosis. We found that patients carrying rs886277 C allele and CC genotype had an increased risk of liver fibrosis progression and cirrhosis development. The association found between *TRPM5* rs886277 polymorphism and liver fibrosis and cirrhosis was independent of the effect of other SNPs, since logistic regression models were adjusted by the most relevant covariables, including five SNPs previously reported in this cohort (*MERTK* rs4374383 [37], *PNPLA3* rs738409 [38], *IL7RA* rs6897932 [35], *MTHFR* rs1801133 [39], and *DARC* rs12075 [30]). These five SNPs were related to liver fibrosis progression and development of cirrhosis [30,35,37,38,39].

TRPM5 is a Ca^2+^-impermeable channel that modulates cellular Ca^2+^ entry, determines the membrane potential, and regulates nerve signals and insulin secretion [14,15]. In a negative feedback loop, Ca^2+^ activates TRPM5 to promote Na^+^ influx, which induces membrane depolarization and a subsequent decrease in the driving force for Ca^2+^ entry [14,42,43]. TRPM5 is present in pancreatic β-cells, where it modulates glucose metabolism. Glucose-induced insulin secretion is decreased and glucose tolerance is impaired in Trpm5−/− mice [44], while activation of TRPM5 may stimulate the pancreatic β-cells to secrete insulin, preventing the onset of diabetes mellitus type II [45,46]. Minor alleles of several *TRPM5* SNPs, which are in linkage disequilibrium with rs886277, have been related to higher glucose level and reduced insulin sensitivity during an oral glucose tolerance test [47] and metabolic syndrome [48]. These two factors are associated with the development of steatosis, hepatic fibrosis, and cirrhosis [49]. On the other hand, calcium is a secondary messenger that regulates multiple hepatic functions, and its dysregulation is a hallmark of chronic liver diseases, which may also hinder liver regeneration [50]. *TRPM5* rs886277 polymorphism is a missense variant (Asn235Ser) in exon 5, which could generate a protein with altered expression or channel functions, causing an increase in intracellular Ca^2+^ and hepatotoxicity, resulting in hepatic scarring and cirrhosis.

We explore the putative functionality of TRPM5 rs886277 polymorphism with the rVarBase database [51]. We observed that this variant is located in an active chromatin region, which could be contributing to gene expression changes. In fact, this has been described in primary natural killer (NK) cells from peripheral blood. In the liver, NK cells account for almost 50% of all intrahepatic lymphocytes, playing a critical role in regulating the liver immune response in both physiological and pathological circumstances [52]. In this setting, *TRPM5* rs886277 polymorphism could lead to altered gene transcription in NK cells, contributing to liver disease’s pathogenesis. Additionally, an analysis of TRPM5 rs886277 polymorphism in the Genotype-Tissue Expression (GTEx) Portal [53], a public resource that provides data of tissue-specific gene expression and regulation according to variant data, showed this polymorphism had been described as an expression quantitative trait loci (eQTL), the C allele and CC genotype being linked to lower TRPM5 expression in pancreas. Moreover, since a sustained inflammatory response is involved in liver injury, it is interesting to note that TRPM5 deficiency in mice increases inflammatory cytokine production in B lymphocytes following lipopolysaccharide stimulation and exacerbates endotoxic shock severity [42]. These studies suggest that defects in the expression or functionality of TPRM5 may promote a sustained inflammatory response contributing to fibrosis progression and cirrhosis development.

## 5. Limitations of the Study

Firstly, our study has a retrospective design and may introduce determination and selection biases. Furthermore, the retrospective design has also led to the absence of relevant clinical data to assess liver disease progression. Secondly, the sample size was small, which limited statistical power. Thirdly, the follow-up time was variable in each patient, but all the patients included in the study had more than 12 months of follow-up (75% had more than 28 months), and it was similar among *TRPM5* rs886277 genotypes. Finally, more than 20% of patients were non-responders to previous interferon therapy. However, we decided to include them because IFN-based treatment does not seem to protect against the progression of CHC in non-responders [54].

## 6. Conclusions

*TRPM5* rs886277 polymorphism was associated with liver fibrosis progression and cirrhosis development among HCV-infected patients. Specifically, the *TRPM5* rs886277 C allele and CC genotype were risk factors in the progression of liver fibrosis and cirrhosis compared to the *TRPM5* rs886277 T allele and TT/CT genotype, respectively.

## Figures and Tables

**Table 1:** Clinical and epidemiological characteristics of HCV-infected patients at baseline.

	*TRPM5* rs886277 Polymorphism			
Characteristic	TT	CT	CC	*p*-Value
**No.**	85	95	28	
**Male**	41 (48.2%)	51 (53.7%)	20 (71.4%)	0.102
**Age (years)**	47.5 (41.3; 59.3)	46.6 (41; 56.1)	46.7 (43.4; 58.3)	0.958
**Time of HCV infection (years)**	7.7 (3.5; 12.9)	9.5 (3.3; 13.8)	6.2 (1.4; 11.6)	0.284
**High alcohol intake**	12 (14.1%)	11 (11.6%)	5 (17.9%)	0.675
**Prior injection drug use**	12 (14.1%)	7 (7.4%)	2 (7.1%)	0.278
**HCV genotype (n = 204)**				
**1**	66 (77.6%)	83 (90.2%)	25 (92.6%)	0.032
**3**	7 (8.2%)	6 (6.5%)	1 (3.7%)	0.709
**4**	11 (12.9%)	3 (3.3%)	1 (3.7%)	0.035
**5**	1 (1.2%)	-	-	-
**Prior failed IFN therapy**	18 (21.2%)	24 (25.3%)	5 (17.9%)	0.656
**Baseline LSM (kPa)**	6.3 (5.2; 7.8)	5.9 (4.9; 7)	6.7 (5.4; 9)	0.328
**F0–F1 (<7.1 kPa)**	58 (68.2%)	73 (76.8%)	18 (64.3%)	0.287
**F2 (7.1–9.4 kPa)**	17 (20%)	15 (15.8%)	6 (21.4%)	0.725
**F3 (9.5–12.4 kPa)**	10 (11.8%)	7 (7.4%)	4 (14.3%)	0.255

Statistics: values were expressed as absolute numbers (%) or median (percentile 25; percentile 75). *p*-values were calculated with the Kruskal–Wallis test for continuous variables or Chi-square test for categorical variables. Abbreviations: HCV, hepatitis C virus; LSM, liver stiffness measurement; kPa, kilopascal; IFN, interferon; *TRPM5*, transient receptor potential cation channel subfamily M member 5.

**Table 2:** Summary of characteristics of *TRPM5* rs886277 polymorphism in patients infected with HCV compared to the Iberian population (data from 1000 Genomes Project Phase 3) [41].

		HCV Cohort	IBS Group	*p*-value
**No.**		208	107	
**Alleles**	**C**	151 (36.3%)	80 (37.4%)	0.367
	**T**	265 (63.7%)	134 (62.6%)	
**Genotype**	**CC**	28 (13.4%)	17 (15.9%)	0.816
	**CT**	95 (45.7%)	46 (43.0%)	
	**TT**	85 (40.9%)	44 (41.1%)	
**HWE (*p*-value)**		0.858	0.398	

Statistics: Values expressed as absolute numbers (%). *p*-values were calculated by the Chi-squared test. Abbreviations: HCV, hepatitis C virus; IBS, Iberian populations in Spain; HWE, Hardy–Weinberg equilibrium; *TRPM5*, transient receptor potential cation channel subfamily M member 5.

**Table 3:** Association between *TRPM5* rs886277 polymorphism and liver fibrosis progression during the follow-up in HCV-infected patients.

	Unadjusted		Adjusted	
Outcome	AMR (95% CI)	*p* ^(a)^	aAMR (95% CI)	*p* ^(b)^
**LSM2/LSM1**				
Additive (CC vs. CT vs. TT)	1.15 (1.05; 1.25)	0.002	1.08 (0.99; 1.17)	0.061
Recessive (CC vs. TT/CT)	1.44 (1.20; 1.72)	<0.001	1.31 (1.12; 1.55)	0.001
				
**Progression to F4**	**OR (95% CI)**	***p*^(a)^**	**aOR (95% CI)**	***p*^(b)^**
Additive (CC vs. CT vs. TT)	1.91 (1.06; 3.45)	0.032	2.64 (1.21; 5.75)	0.014
Recessive (CC vs. TT/CT)	2.82 (1.06; 7.51)	0.038	4.33 (1.18; 15.91)	0.027

Statistics: *p*-values were calculated by univariate regression (a) and multivariate regression (b) adjusted by the most relevant covariates (see statistical analysis section). Significant differences are shown in bold. Abbreviations: aAMR, adjusted arithmetic mean ratio; aOR, adjusted odds ratio; 95%CI, 95% confidence interval; *p*-value, level of significance; LSM, liver stiffness measurement; F4, cirrhosis; *TRPM5*, transient receptor potential cation channel subfamily M member 5.

## Data Availability

The data set used for this study may be made available by the corresponding author upon reasonable request.

## Abbreviations

Chronic hepatitis C	CHC
Hepatitis C virus	HCV
Direct-acting antivirals	DAAs
Single nucleotide polymorphisms	SNPs
Transient receptor potential cation channel subfamily M member 5	TRPM5
Cirrhosis risk score	CRS
Liver stiffness measurement	LSM
Cirrhosis	F4; LSM1 ≥ 12.5 kPa
Kilopascals	kPa
Baseline LSM	LSM1
Final LSM	LSM2
Generalized linear models	GLM
Arithmetic mean ratio	AMR
Odds ratio	OR
Statistical Package for the Social Sciences	SPSS
Iberian population in Spain	IBS
Natural killer	NK

## References

[1] The Polaris Observatory HCV Collaborators (2017). Global prevalence and genotype distribution of hepatitis C virus infection in 2015: A modelling study. Lancet Gastroenterol. Hepatol..

[2] Stanaway J.D., Flaxman A.D., Naghavi M., Fitzmaurice C., Vos T., Abubakar I., Abu-Raddad L.J., Assadi R., Bhala N., Cowie B. (2016). The global burden of viral hepatitis from 1990 to 2013: Findings from the Global Burden of Disease Study 2013. Lancet.

[3] World Health Organization (2017). Global Hepatitis Report 2017.

[4] Lingala S., Ghany M.G. (2015). Natural History of Hepatitis C. Gastroenterol. Clin. N. Am..

[5] Conti F., Buonfiglioli F., Scuteri A., Crespi C., Bolondi L., Caraceni P., Foschi F.G., Lenzi M., Mazzella G., Verucchi G. (2016). Early occurrence and recurrence of hepatocellular carcinoma in HCV-related cirrhosis treated with direct-acting antivirals. J. Hepatol..

[6] Forner A.R., Bruix J. (2018). Hepatocellularcarcinoma. Lancet.

[7] Aghemo A., Berenguer M., Dalgard O., Dusheiko G., Marra F., Puoti M., Wedemeyer H. (2020). EASL recommendations on treatment of hepatitis C: Final update of the series. J. Hepatol..

[8] Castera L. (2015). Noninvasive Assessment of Liver Fibrosis. Dig. Dis..

[9] Resino S., Sánchez-Conde M., Berenguer J. (2012). Coinfection by human immunodeficiency virus and hepatitis C virus: Noninvasive assessment and staging of fibrosis. Curr. Opin. Infect. Dis..

[10] Castera L. (2011). Invasive and non-invasive methods for the assessment of fibrosis and disease progression in chronic liver disease. Best Prac. Res. Clin. Gastroenterol..

[11] Patel K., Sebastiani G. (2020). Limitations of non-invasive tests for assessment of liver fibrosis. JHEP Rep..

[12] Rüeger S., Bochud P., Dufour J.-F., Müllhaupt B., Semela D., Heim M., Moradpour D., Cerny A., Malinverni R., Booth D. (2015). Impact of common risk factors of fibrosis progression in chronic hepatitis C. Gut.

[13] Heim M.H., Bochud P.-Y., George J. (2016). Host–hepatitis C viral interactions: The role of genetics. J. Hepatol..

[14] Prawitt D., Monteilh-Zoller M.K., Brixel L., Spangenberg C., Zabel B., Fleig A., Penner R. (2003). TRPM5 is a transient Ca2+-activated cation channel responding to rapid changes in [Ca^2+^]i. Proc. Natl. Acad. Sci. USA.

[15] Liu D., Liman E.R. (2003). Intracellular Ca2+ and the phospholipid PIP2 regulate the taste transduction ion channel TRPM5. Proc. Natl. Acad. Sci. USA.

[16] Luo X.C., Chen Z.H., Xue J.B., Zhao D.X., Lu C., Li Y.H., Li S.M., Du Y.W., Liu Q., Wang P. (2019). Infection by the parasitic helminth Trichinella spiralis activates a Tas2r-mediated signaling pathway in intestinal tuft cells. Proc. Natl. Acad. Sci. USA.

[17] Maina I.W., Workman A.D., Cohen N.A. (2018). The role of bitter and sweet taste receptors in upper airway innate immunity: Recent advances and future directions. World J. Otorhinolaryngol. Head Neck Surg..

[18] O’Leary C.E., Schneider C., Locksley R.M. (2019). Tuft Cells-Systemically Dispersed Sensory Epithelia Integrating Immune and Neural Circuitry. Ann. Rev. Immunol..

[19] Perniss A., Liu S., Boonen B., Keshavarz M., Ruppert A.L., Timm T., Pfeil U., Soultanova A., Kusumakshi S., Delventhal L. (2020). Chemosensory Cell-Derived Acetylcholine Drives Tracheal Mucociliary Clearance in Response to Virulence-Associated Formyl Peptides. Immunity.

[20] Rane C.K., Jackson S.R., Pastore C.F., Zhao G., Weiner A.I., Patel N.N., Herbert D.R., Cohen N.A., Vaughan A.E. (2019). Development of solitary chemosensory cells in the distal lung after severe influenza injury. Am. J. Physiol. Lung Cell Mol. Physiol..

[21] Saunders C.J., Christensen M., Finger T.E., Tizzano M. (2014). Cholinergic neurotransmission links solitary chemosensory cells to nasal inflammation. Proc. Natl. Acad. Sci. USA.

[22] Tizzano M., Gulbransen B.D., Vandenbeuch A., Clapp T.R., Herman J.P., Sibhatu H.M., Churchill M.E., Silver W.L., Kinnamon S.C., Finger T.E. (2010). Nasal chemosensory cells use bitter taste signaling to detect irritants and bacterial signals. Proc. Natl. Acad. Sci. USA.

[23] Fernandez-Rodriguez A., Berenguer J., Jimenez-Sousa M.A., Guzman-Fulgencio M., Micheloud D., Miralles P., Lopez J.C., Bellon J.M., Aldamiz-Echevarria T., Garcia-Broncano P. (2013). Prediction of hepatic fibrosis in patients coinfected with HIV and hepatitis C virus based on genetic markers. J. Acquir. Immun. Defic. Syndr..

[24] Marcolongo M., Young B., Dal Pero F., Fattovich G., Peraro L., Guido M., Sebastiani G., Palu G., Alberti A. (2009). A seven-gene signature (cirrhosis risk score) predicts liver fibrosis progression in patients with initially mild chronic hepatitis C. Hepatology.

[25] Trepo E., Potthoff A., Pradat P., Bakshi R., Young B., Lagier R., Moreno C., Verset L., Cross R., Degre D. (2011). Role of a cirrhosis risk score for the early prediction of fibrosis progression in hepatitis C patients with minimal liver disease. J. Hepatol..

[26] Huang H., Shiffman M.L., Friedman S., Venkatesh R., Bzowej N., Abar O.T., Rowland C.M., Catanese J.J., Leong D.U., Sninsky J.J. (2007). A 7 gene signature identifies the risk of developing cirrhosis in patients with chronic hepatitis C. Hepatology.

[27] Curto T.M., Lagier R.J., Lok A.S., Everhart J.E., Rowland C.M., Sninsky J.J., The H.-C.T.g. (2011). Predicting cirrhosis and clinical outcomes in patients with advanced chronic hepatitis C with a panel of genetic markers (CRS7). Pharmacog. Genom..

[28] Li Y., Chang M., Abar O., Garcia V., Rowland C., Catanese J., Ross D., Broder S., Shiffman M., Cheung R. (2009). Multiple variants in toll-like receptor 4 gene modulate risk of liver fibrosis in Caucasians with chronic hepatitis C infection. J. Hepatol..

[29] Zimmermann A., Darstein F., Hoppe-Lotichius M., Toenges G., Lautem A., Abel F., Schad A., Mittler J., Vollmar J., Grimm D. (2019). Cirrhosis risk score of the donor organ predicts early fibrosis progression after liver transplantation. J. Gastrointestin. Liver Dis..

[30] Jiménez-Sousa M.Á., Gómez-Moreno A.Z., Pineda-Tenor D., Sánchez-Ruano J.J., Artaza-Varasa T., Martin-Vicente M., Fernández-Rodríguez A., Martínez I., Resino S. (2019). Impact of DARC rs12075 Variants on Liver Fibrosis Progression in Patients with Chronic Hepatitis C: A Retrospective Study. Biomolecules.

[31] Calvaruso V., Craxì A. (2012). 2011 E uropean A ssociation of the S tudy of the L iver hepatitis C virus clinical practice guidelines. Liver International..

[32] EASL (2014). Clinical Practice Guidelines: Management of hepatitis C virus infection. J. Hepatol..

[33] Spanish National Genotyping Center. http://www.cegen.org.

[34] Gabriel S., Ziaugra L., Tabbaa D. (2009). SNP genotyping using the Sequenom MassARRAY iPLEX platform. Curr. Protoc. Hum. Genet..

[35] Jiménez-Sousa M.Á., Gómez-Moreno A.Z., Pineda-Tenor D., Medrano L.M., Sánchez-Ruano J.J., Fernández-Rodríguez A., Artaza-Varasa T., Saura-Montalbán J., Vazquez-Moron S., Ryan P. (2018). The IL7RA rs6897932 polymorphism is associated with progression of liver fibrosis in patients with chronic hepatitis C: Repeated measurements design. PLoS ONE.

[36] Castera L., Forns X., Alberti A. (2008). Non-invasive evaluation of liver fibrosis using transient elastography. J. Hepatol..

[37] Jiménez-Sousa M.Á., Gómez-Moreno A.Z., Pineda-Tenor D., Brochado-Kith O., Sánchez-Ruano J.J., Artaza-Varasa T., Gómez-Sanz A., Fernández-Rodríguez A., Resino S. (2018). The myeloid-epithelial-reproductive tyrosine kinase (MERTK) rs4374383 polymorphism predicts progression of liver fibrosis in hepatitis C virus-infected patients: A longitudinal study. J. Clin. Med..

[38] Jiménez-Sousa M.Á., Gómez-Moreno A.Z., Pineda-Tenor D., Sánchez-Ruano J.J., Fernández-Rodríguez A., Artaza-Varasa T., Gómez-Sanz A., Martín-Vicente M., Vázquez-Morón S., Resino S. (2018). PNPLA3 rs738409 polymorphism is associated with liver fibrosis progression in patients with chronic hepatitis C: A repeated measures study. J. Clin. Virol..

[39] Pineda-Tenor D., Gomez-Moreno A.Z., Sanchez-Ruano J.J., Artaza-Varasa T., Virseda-Berdices A., Fernandez-Rodriguez A., Mendoza P.M., Jimenez-Sousa M.A., Resino S. (2020). MTHFR rs1801133 Polymorphism Is Associated with Liver Fibrosis Progression in Chronic Hepatitis C: A Retrospective Study. Front. Med..

[40] 1000 Genomes Project. http://www.1000genomes.org/home.

[41] 1000 Genomes Project Phase 3. http://grch37.ensembl.org/index.html.

[42] Sakaguchi T., Okumura R., Ono C., Okuzaki D., Kawai T., Okochi Y., Tanimura N., Murakami M., Kayama H., Umemoto E. (2020). TRPM5 Negatively Regulates Calcium-Dependent Responses in Lipopolysaccharide-Stimulated B Lymphocytes. Cell Rep..

[43] Hofmann T., Chubanov V., Gudermann T., Montell C. (2003). TRPM5 is a voltage-modulated and Ca(2+)-activated monovalent selective cation channel. Curr. Biol..

[44] Colsoul B., Schraenen A., Lemaire K., Quintens R., Van Lommel L., Segal A., Owsianik G., Talavera K., Voets T., Margolskee R.F. (2010). Loss of high-frequency glucose-induced Ca^2+^ oscillations in pancreatic islets correlates with impaired glucose tolerance in Trpm5-/- mice. Proc. Natl. Acad. Sci. USA.

[45] Philippaert K., Pironet A., Mesuere M., Sones W., Vermeiren L., Kerselaers S., Pinto S., Segal A., Antoine N., Gysemans C. (2017). Steviol glycosides enhance pancreatic beta-cell function and taste sensation by potentiation of TRPM5 channel activity. Nat. Commun..

[46] Islam M.S. (2020). Molecular Regulations and Functions of the Transient Receptor Potential Channels of the Islets of Langerhans and Insulinoma Cells. Cells.

[47] Ketterer C., Mussig K., Heni M., Dudziak K., Randrianarisoa E., Wagner R., Machicao F., Stefan N., Holst J.J., Fritsche A. (2011). Genetic variation within the TRPM5 locus associates with prediabetic phenotypes in subjects at increased risk for type 2 diabetes. Metabolism.

[48] Tabur S., Oztuzcu S., Duzen I.V., Eraydin A., Eroglu S., Ozkaya M., Demiryurek A.T. (2015). Role of the transient receptor potential (TRP) channel gene expressions and TRP melastatin (TRPM) channel gene polymorphisms in obesity-related metabolic syndrome. Eur. Rev. Med. Pharmacol. Sci..

[49] Moon A.M., Singal A.G., Tapper E.B. (2020). Contemporary Epidemiology of Chronic Liver Disease and Cirrhosis. Clin. Gastroenterol. Hepatol..

[50] Oliva-Vilarnau N., Hankeova S., Vorrink S.U., Mkrtchian S., Andersson E.R., Lauschke V.M. (2018). Calcium Signaling in Liver Injury and Regeneration. Front. Med..

[51] Guo L., Du Y., Qu S., Wang J. (2016). rVarBase: An updated database for regulatory features of human variants. Nucleic Acids Res..

[52] Mikulak J., Bruni E., Oriolo F., Di Vito C., Mavilio D. (2019). Hepatic Natural Killer Cells: Organ-Specific Sentinels of Liver Immune Homeostasis and Physiopathology. Front. Immunol..

[53] Genotype-Tissue Expression (GTEx) Portal. https://gtexportal.org.

[54] Carmona I., Cordero P., Ampuero J., Rojas A., Romero-Gomez M. (2016). Role of assessing liver fibrosis in management of chronic hepatitis C virus infection. Clin. Microbiol. Infect..

